# Automatically Controlled Dust Generation System Using Arduino †

**DOI:** 10.3390/s22124574

**Published:** 2022-06-17

**Authors:** Dan Hofstetter, Eileen Fabian, Dorian Dominguez, A. Gino Lorenzoni

**Affiliations:** 1 Department of Agricultural and Biological Engineering, The Pennsylvania State University, University Park, PA 16802, USA; efw2@psu.edu; 2 Department of Agricultural and Biological Engineering, University of Florida, Gainesville, FL 32611, USA; 3 Department of Animal Science, The Pennsylvania State University, University Park, PA 16802, USA; dxd5537@psu.edu (D.D.); agl20@psu.edu (A.G.L.)

**Keywords:** dust generator, Arduino, dust sensor, airborne dust concentration

## Abstract

A dust generator was developed to disperse and maintain a desired concentration of airborne dust in a controlled environment chamber to study poultry physiological response to sustained elevated levels of particulate matter. The goal was to maintain an indicated PM10 concentration of 50 µg/m^3^ of airborne dust in a 3.7 m × 4.3 m × 2.4 m (12 ft × 14 ft × 8 ft) controlled environment chamber. The chamber had a 1.5 m^3^/s (3200 cfm) filtered recirculation air handling system that regulated indoor temperature levels and a 0.06 m^3^/s (130 cfm) exhaust fan that exchanged indoor air for fresh outdoor air. Dry powdered red oak wood dust that passed through an 80-mesh screen cloth was used for the experiment. The dust generator metered dust from a rectangular feed hopper with a flat bottom belt to a 0.02 m^3^/s (46 cfm) centrifugal blower. A vibratory motor attached to the hopper ran only when the belt was operated to prevent bridging of powdered materials and to provide an even material feed rate. A laser particle counter was used to measure the concentration of airborne dust and provided feedback to an Arduino-based control system that operated the dust generator. The dust generator was operated using a duty cycle of one second on for every five seconds off to allow time for dispersed dust to mix with chamber air and reach the laser particle counter. The control system maintained an airborne PM10 dust concentration of 54.92 ± 6.42 µg/m^3^ in the controlled environment chamber during six weeks of continuous operation using red oak wood dust. An advantage of the automatically controlled dust generator was that it continued to operate to reach the setpoint concentration in response to changes in material flow due to humidity, partial blockages, and non-uniform composition of the material being dispersed. Challenges included dust being trapped by the recirculation filter and the exhaust fan removing airborne dust from the environmental chamber.

## 1. Introduction

Animals raised in barns are regularly exposed to varying levels of airborne dust (particulate matter). It is well known that poultry dust affects bird health [1,2,3], especially their respiratory function [4,5,6,7,8,9,10,11,12,13,14]. However, the impact of specific types of particulate matter is not well understood. This is because “poultry dust” is composed of many different organic particles including manure, feed, feathers, dander [4,5,10], and inorganic materials from barn construction such as wood, insulation, and concrete [5]. Particulate matter is also described by its size, especially in relation to its ability to penetrate deep into the respiratory system [15]. Dust can also absorb water-soluble gases, such as ammonia [10,16], which brings the gases into close contact with tissues deeper in the lungs and causes lesions in respiratory epithelia [8,16].

There are challenges to studying dust exposure effects for animals living in barns due to uncontrolled conditions. Ventilation rates, type of bedding used, animal stocking density, animal age and circadian patterns affect dust concentrations inside the barn [5,17]. Reported respirable dust concentrations in poultry barns range from 0.13 mg/m^3^ for caged laying hen housing to 1.19 mg/m^3^ for hen aviary systems [5,18]. However, these values are highly variable. For example, for poultry raised on litter (a combination of bedding material and manure) the accumulation of manure causes the percentage of original bedding material to decline by the end of the growing period [17], thus the composition of dust the animals are exposed to changes over time, making it difficult to examine the effect of a specific type of dust on animal physiology. Since indoor environmental conditions are difficult to tightly control, and indoor dust levels and composition vary greatly in animal barns [5], controlled studies are needed to determine the effect of dust on the airways of animals.

The goal of this project was to develop a dust generator for poultry health research that could be automatically controlled based on real-time measurements to maintain a steady airborne concentration of dust. The design requirements were: (1) low-cost, easy to source components; (2) hopper capacity large enough to hold dust for 24 h of continuous operation; (3) minimal required maintenance input from personnel; (4) capability to handle a variety of powdered materials; (5) ability to maintain dust concentration within 10% of the setpoint. This manuscript focuses on the components of the control system related to automatic dust generation: the microcontroller, the dust sensor, and the dust generator.

### 1.1. Dust Generation Methods

Commercial dust generators for dispersing airborne particulates exist, but their cost is typically high [19]. In addition, their ability to disperse large amounts of dust is limited by a small dust hopper volume because they are designed for laboratory applications. Many commercial dust generators rely on compressed air to disperse particulate matter, but output concentration is based on material feed rate and not measured airborne concentration.

Dust generators built for research are typically made for a specific purpose, and many are built using modified repurposed equipment [20]. A Wright dust feed [21] consists of a rotating vertical cylinder filled with compacted dust, with a stationary blade that scrapes a thin layer of material into the air stream to generate a dust aerosol [21,22]. Others have retrofitted the Wright dust feed with a microprocessor controlled stepper motor for more precise operator control over rotational speed [23], added automatic control of the stepper motor using a personal computer [24], and developed a feedback control system based on real-time measurements from an aerosol monitor [25]. Limitations of the Wright dust feed have been documented including limited operating speed and a small dust holding capacity. Crider et al. developed a cone-shaped dry powder aerosol dispensing device in the 1960s that used Teflon chips to agitate the bulk powder with a pulsed air jet to maintain concentrations from 0.7 to 3.0 mg/m^3^, and it was capable of automatic feedback control based on measurements from an aerosol photometer [26]. McKinney et al. developed a computer automated acoustical aerosol generator for animal toxicology research that suspended silica particles into the air using vibrations from a loudspeaker and dispersed them using high velocity air flow through a venturi [27]. Domingo et al. developed a drum-type dust generator to produce large volumes of field dust to simulate exposure to soil-derived dusts [28]. Mendez et al. developed a laboratory dust generator with a rotating generation chamber and a stationary concentration chamber made from common plastic bottles, using a vacuum source to aspirate air loaded with fine particulates [19]. There are several restrictions from the listed equipment (commercial and experimental) that prompted us to develop a specific dust generator. First, most available equipment is designed to work with relatively small amounts of dust that are adequate for small environmental chambers. Second, some of the listed equipment required an elaborate conditioning of the dust (compacting) before its use. Third, most of the commercially available equipment is expensive.

### 1.2. Dust Sensing Technology

Many types of dust sensors that can be used to measure the concentration of airborne particulate matter are commercially available [29,30]. Most sensors work on the principle of light scattering, where a beam of light is emitted through a sample of air containing particulates, and the particulates reflect, or scatter, some of the light beam to a photosensor that measures light intensity [29,31,32]. The intensity of the reflected light is then correlated to the particle count in the air sample [32,33]. This particle count is often expressed in units of density (µg/m^3^ or mg/m^3^ commonly), where the density of the sample is inferred based on calibration using “standard particles” with a very uniform spherical shape, diameter, and mass [32].

Analytical instruments such as the DustTrak II model 8530 (TSI Inc., Shoreview, Minnesota, USA) have been widely used in field studies to measure airborne dust concentrations in animal barns. They are highly accurate, but also have a high cost (around $6000 USD), which makes deployment of multiple units expensive. Handheld instruments such as the MetOne Handheld Particle Counter model HHPC3+ (Beckman Coulter Life Sciences, Indianapolis, IN, USA) can be used to make spot measurements easily. The HHPC3+ has three measurement “bins” or sizes of particles that can be counted and recorded at one time. The cost of the HHPC3+ is around $3000 USD.

Many low-cost, consumer grade dust sensors are available. Some, such as the Sharp GP2Y1010A0F or Shinyei PPD42 use an infrared LED as the light source and passively sample nearby air by use of a heated resistor that causes convection. Holes in the sensor housing allow sample air to enter below the heated resistor, and the rising air current travels past the beam of light, with particulate matter scattering some of the light to a phototransistor. The PPD42 sensors have been shown to be accurate at detecting particles in the range of 0.75 to 6 µm [30], but are not good at detecting particles smaller than 0.5 µm. This type of sensor also has a response time on the order of minutes, making it suitable for trend tracking but less useful for real-time aerosol generator control. There are also inexpensive (cost around $50 USD) active sampling optical particle counters available such as the Plantower PMS5003 (Plantower, Beijing, China) that utilize laser diodes as a light source and axial fans to draw a constant rate of sample air through the sensing chamber. Many comparisons to higher cost reference instruments have reported good performance and build quality of the PMS5003 sensor [29]. The PMS5003 was found to have a very low limit of detection without exhibiting temporal lag time [32], making the PMS5003 suitable for real-time measurements of small particles.

## 2. Materials and Methods

### 2.1. Air Quality Monitor and Datalogger

A custom-built indoor air quality monitor logged data and controlled the concentrations of airborne dust in the chamber [34]. An Arduino MEGA 2560 microcontroller (Elegoo, Inc., Shenzhen, China) recorded temperature, relative humidity, luminosity, carbon dioxide concentration, ammonia gas concentration, dust concentration, and exhaust air speed. A real-time clock module with backup battery provided the time and date. All measured data were logged to a 16GB microSD card using a comma-separated value (.CSV) file format that could be opened using Microsoft Excel. A 2.4 cm (0.96 in.) LCD display showed measured temperature, humidity, and ammonia, carbon dioxide, and dust concentrations. A Wi-Fi module was installed to allow data to be sent to a website for real-time online monitoring of the controlled indoor environment.

### 2.2. Dust Sensor

Airborne dust concentration was monitored using a Plantower PMS5003 laser particle counter (dust sensor) connected to the Arduino microcontroller. The PMS5003 was selected because it actively draws sample air into the sensor housing using an axial fan, could detect particles with a diameter as small as 0.3 µm, and could utilize hardware serial communication with the Arduino microcontroller.

**Figure 1:**
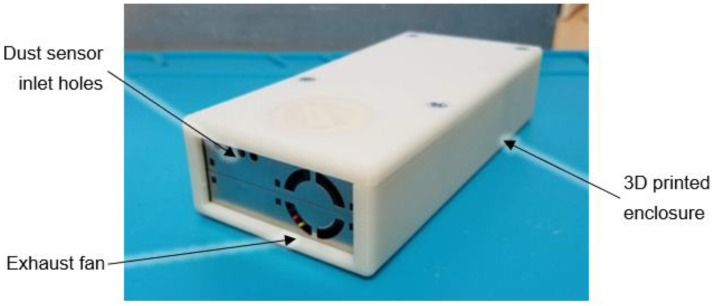
PMS5003 dust sensor mounted in 3D printed enclosure.

**Figure 2:**
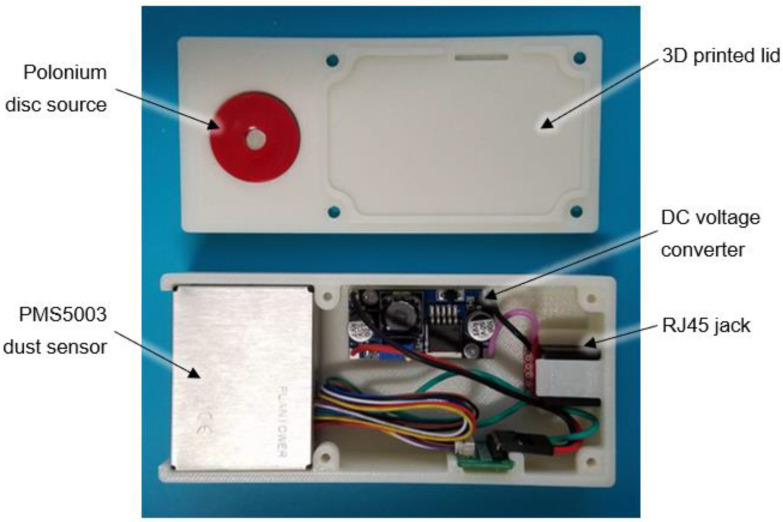
Inside view of 3D printed dust sensor enclosure showing components.

The sensor was housed in a custom designed 3D printed enclosure (Figure 1). Air was drawn into the dust sensor through four small holes in the upper left corner of the sensor housing and exhausted by a small axial fan. An ethernet cable connected to the datalogger provided 12 VDC power that was converted to 5 VDC in the dust sensor enclosure. The lid of the enclosure contained a Polonium-210 disc source (United Nuclear Scientific, Klamath Falls, OR, USA) that emitted alpha particles to reduce nearby static charge and prevent dust from collecting on interior plastic surfaces of the dust sensor. Figure 2 is an inside view of the 3D printed enclosure showing the components.

Dust concentration data that were logged included PM1.0, PM2.5, and PM10 concentrations (µg/m^3^), and counts per unit volume for particle sizes of 0.3, 0.5, 1.0, 2.5, 5.0, and 10.0 microns (pcs/0.1 L). The indicated PM10 concentration from the PMS5003 sensor was used for dust generator control.

### 2.3. Conveyor Body

**Figure 3:**
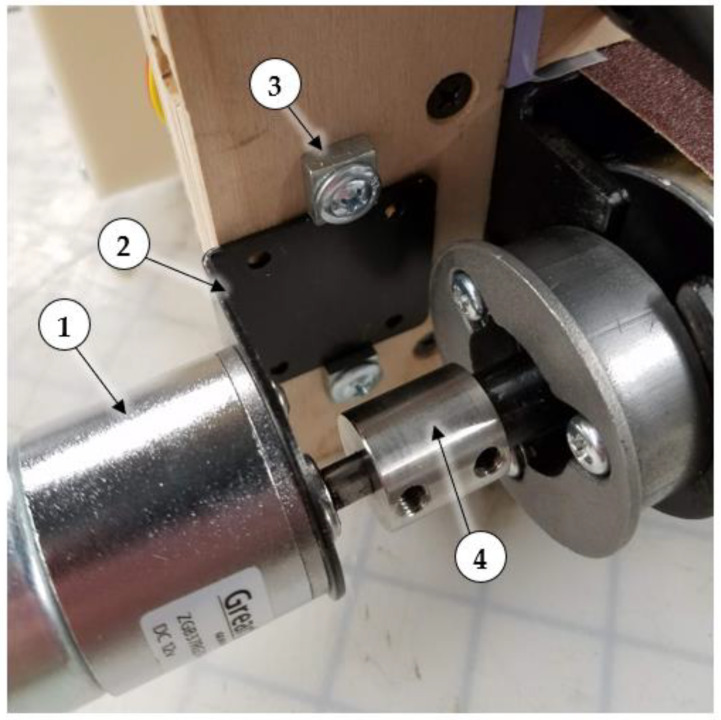
Assembled (1) gearmotor, (2) L-shaped torque arm bracket, (3) torque arm stops, and (4) 12 mm to 6 mm shaft coupling.

The dust generator was built using the conveyor frame from a 0.1 m × 0.9 m (4 in. × 36 in.) Ryobi BD4601G belt sander (Ryobi, Anderson, SC, USA; $159 USD at Home Depot). The sanding belt frame was removed from the sanding machine and the drive pulley was powered using a 3.5 rpm 12 VDC gearmotor. The gearmotor output shaft was attached to the conveyor drive pulley shaft using a 12 mm to 6 mm shaft coupling. Since the shaft coupling was rigid and did not allow for any misalignment, the gearmotor body was mounted to a 37 mm (1.5 in.) L-shaped bracket that acted as a torque arm to prevent the motor body from rotating while remedying shaft misalignment problems that may occur. Torque arm stops were made from 1/4 in.–20 square nuts fastened to a plywood block on the side of the conveyor using wood screws. Figure 3 shows the assembled (1) gearmotor, (2) L-shaped torque arm bracket, (3) torque arm stops, and (4) shaft coupling.

**Figure 4:**
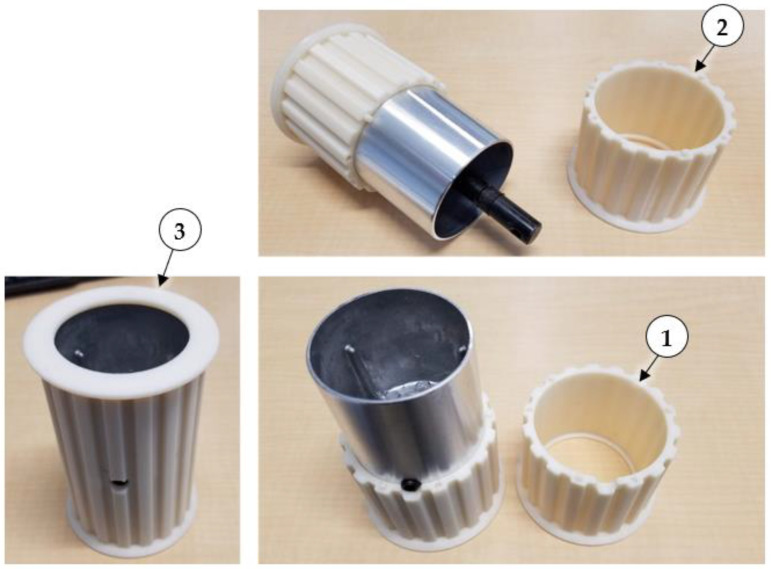
Split mating halves of 3D printed (1) drive and (2) idler pulleys, and (3) drive pulley halves assembled and glued onto aluminum drive pulley.

In preliminary tests, the included 80-grit sanding belt was used to convey dust from a hopper, and dust was aerosolized by a DC blower directed across the top of the belt. However, the sanding belt did not track well and would regularly get stuck against one side of the dust hopper. Instead, a heavy-duty rubber 375H400 timing belt (D&D Global, Rock Valley IA, USA) was used because it could be positively driven via a toothed pulley, and because timing belt pulleys have raised flanges that help to center the belt during operation. Since no suitable 10 cm (4 in.) wide timing pulley was available, custom 18-tooth timing belt pulley sleeves were 3D printed using ABS plastic filament. The Ryobi drive and idler pulleys had different dimensions, so the custom pulley sleeves were designed with the same outer tooth and flange dimensions but different inside dimensions so they would fit tightly onto the original aluminum pulleys. The sleeves were 3D printed as split mating halves that fit over the tapered aluminum Ryobi drive and idler pulleys, and the halves were permanently attached using adhesive (Figure 4). The drive pulley sleeve included a hole to allow access to the setscrew used to secure the aluminum drive pulley to the steel drive shaft.

**Figure 5:**
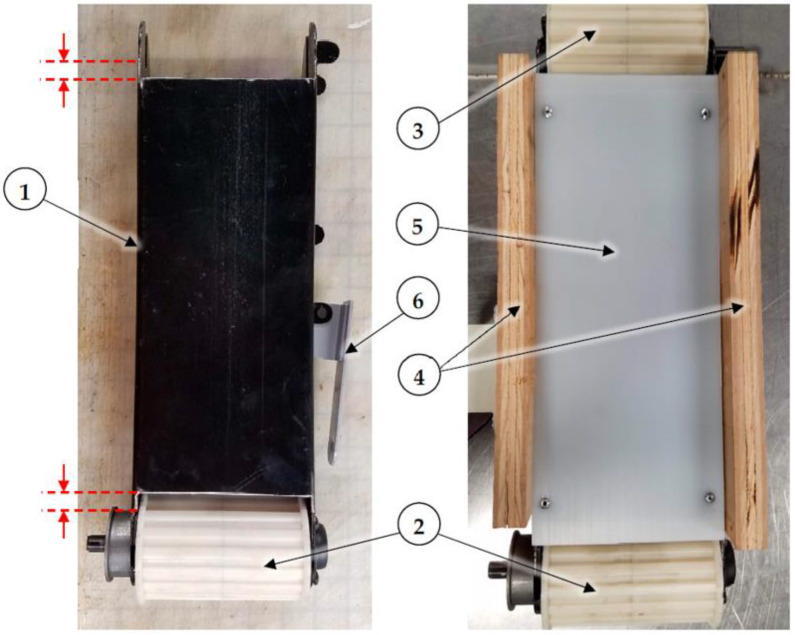
Top view of dust generator showing material removed from (1) conveyor frame between red dashed lines for pulley flange clearance; installed 3D printed (2) drive and (3) idler pulleys; (4) plywood sides; and (5) UHMW liner. Item (6) is the belt tension release lever.

A 1.6 mm (0.06 in.) thick ultra-high molecular weight (UHMW) polyethylene liner was attached to the steel conveyor frame to reduce friction between the rubber belt and the conveyor slider bed. Approximately 12.7 mm (0.5 in.) of material had to be removed from each end of the conveyor frame slider bed to create clearance for the flanged 3D printed pulley sleeves. Figure 5 shows the modified (1) conveyor frame, where the red dashed lines show where material was removed for pulley flange clearance; also shown are the installed 3D printed (2) drive and (3) idler pulleys; (4) plywood sides; (5) UHMW liner; and (6) tension release lever.

**Figure 6:**
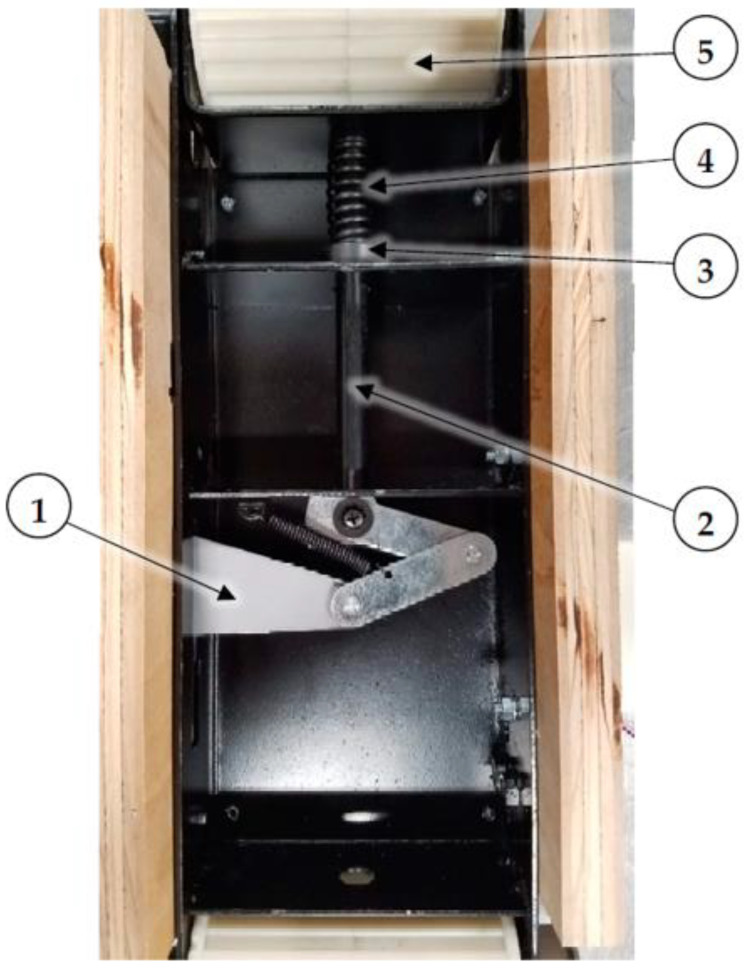
Bottom view of dust generator showing (1) belt tension release lever, (2) connecting link, (3) spacer, (4) spring, and (5) idler pulley (shown for reference).

The sanding machine had a spring-loaded belt tensioning mechanism. Belt tension could be released by a manual lever (item 6 in top view, Figure 5; also, item 1 in bottom view, Figure 6) to allow the original sanding belt to be removed. The stock spring did not exert enough force to remove slack from the heavy-duty rubber timing belt, so the spring was compressed by adding a 12.7 mm (0.5 in.) inside diameter × 15.9 mm (0.6 in.) long shaft collar to the connecting link between the conveyor frame and the original spring. Figure 6 shows (1) the tension release lever, (2) the connecting link, (3) the shaft collar, (4) the spring, and (5) the idler pulley (shown for reference).

### 2.4. Dust Hopper

**Figure 7:**
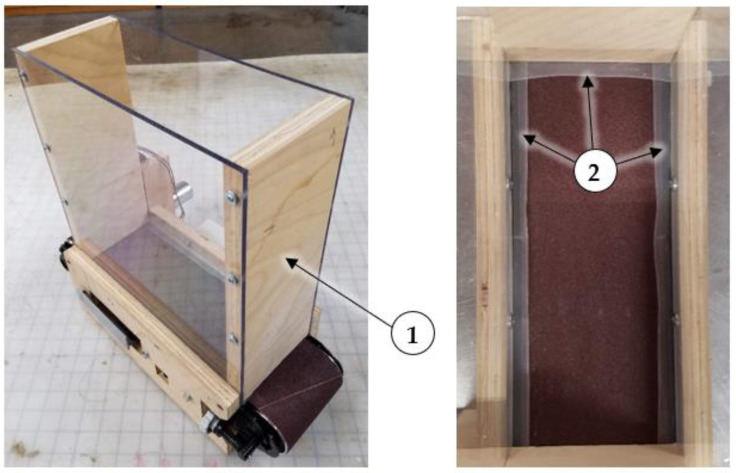
Dust generator with (1) dust hopper, and top view showing (2) side skirts affixed to three sides (note: original sanding belt and pulleys are shown).

A dust hopper was placed on top of the rubber conveyor belt. The hopper was 30 cm high × 27 cm long × 11 cm wide (11.7 in. high × 10.5 in. long × 4.5 in. wide) with plywood ends and clear polycarbonate sides. Dust flowed out of the hopper through a narrow 3.2 mm (0.13 in.) opening under the base of the plywood discharge-end panel. Side skirts were added to the inside of the hopper to prevent dust from spilling out the sides and rear (opposite the discharge end) of the hopper. The side skirts were made from 2.5 cm (1.0 in.) wide adhesive backed UHMW strips. Half of the backing material was left on the strips, and 1.3 cm (0.5 in.) was removed and affixed to the hopper sides. Figure 7 shows the dust generator hopper with side skirts affixed to three sides.

### 2.5. Vibratory Shaker, Hopper Extension, Blower

**Figure 8:**
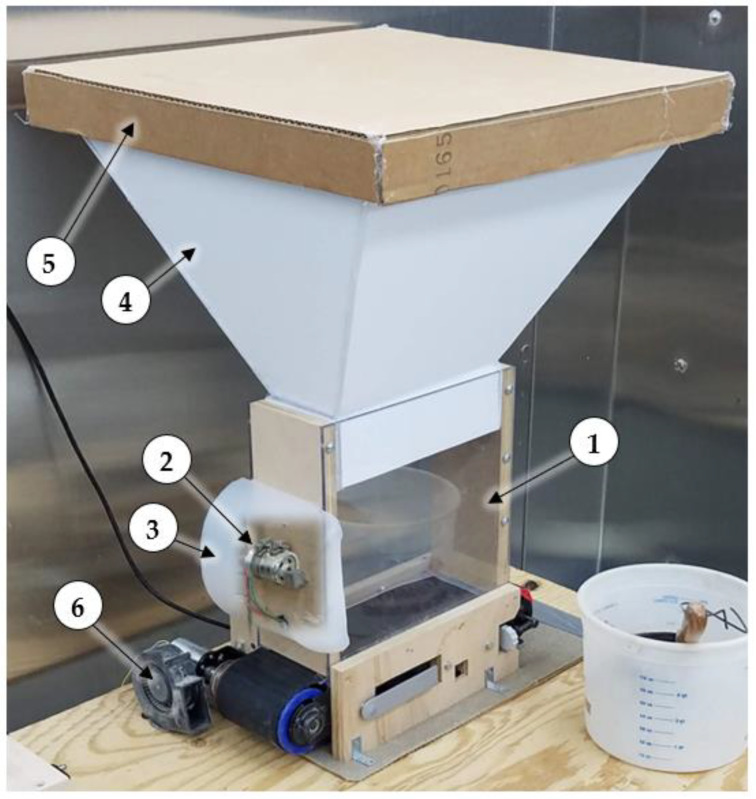
Assembled dust generator: (1) hopper, (2) vibratory motor, (3) safety shield, (4) hopper extension, (5) lid, and (6) centrifugal blower.

A 12 VDC 1800 rpm vibratory shaker motor was mounted to the discharge-end panel of the hopper to prevent material from compacting and bridging above the moving conveyor belt. A relay with a 5 VDC coil switched on 12 VDC power to the vibratory motor when the dust generator gearmotor was operated. A safety shield made from one half of a plastic bottle was glued to the hopper to cover the vibratory motor. A hopper extension was fabricated using foam-core board and mounted to the machine to increase the hopper capacity. A lid was placed on top of the extension to keep out feathers and reduce exposure to humid air to keep the dust dry. Dust exiting the hopper was aerosolized by a centrifugal blower fan (model BFB1012VH-7P03, Delta Electronics, Plano, TX, USA) capable of supplying 0.02 m^3^/s (46 cfm) when operated at 12 VDC. The fan was placed at the end perimeter of the conveyor belt and angled slightly upwards, blowing across the end of the belt such that dust falling off the end of the belt would get blown into the air. The fan was operated continuously using a 9 VDC 1.5 A adapter plugged into a 120 VAC receptacle. Figure 8 shows the assembled dust generator: (1) hopper, (2) vibratory motor, (3) safety shield, (4) hopper extension, (5) lid, and (6) centrifugal blower. A list of parts and electronic components used to build and control the dust generator can be found in Table A1 in Appendix A.

### 2.6. Dust Generator Conveyor Motor Operation

Dust was generated in the controlled environment chamber using the custom-built dust generator powered by a DC gearmotor with a maximum rotational speed of 3.5 rpm. The gearmotor was driven by a L293N motor driver that was controlled by the Arduino MEGA microcontroller. The microcontroller activated the gearmotor when dust levels fell below the desired setpoint. The speed of the gearmotor was capable of being adjusted using pulse-width modulation (PWM) to try to maintain constant levels of dust in the chamber air.

An algorithm was used in the Arduino control code that altered the duty cycle of the gearmotor to operate for 1 out of every 5 s (1 s ON, 4 s OFF), which resulted in a more even delivery of dust into the air and a steadier concentration. The DC blower was run continuously. The vibratory shaker motor functioned only when the conveyor gearmotor was operating. This was accomplished using a 5 VDC (coil) relay that switched on 12 VDC power to the vibratory motor when the dust generator was operated from the L293N controller.

### 2.7. Preliminary Testing

Preliminary testing of the dust generator was performed using several kinds of dust: talcum powder, cornstarch, poultry dust (swept from the barn floor and sieved to remove manure and coarse feathers), wood flour (System Three Resins, Inc., Lacey, WA, USA), and red oak sawdust. Initial dust levels to be maintained were determined experimentally and based on experience of field personnel who entered the controlled environment chamber wearing fitted respirators with P100 filter cartridges. Dust output from the generator was gradually increased, and airborne concentration was evaluated by the personnel using a Handheld Particle Counter (MetOne model HHPC3+) along with a bright flashlight. Dust levels were selected to represent those typically observed in “dusty” commercial poultry facilities. The determined setpoint used for dust generator control was a PM10 level of 50 µg/m^3^ as indicated by the PMS5003 sensor.

### 2.8. Dust Characteristics

**Figure 9:**
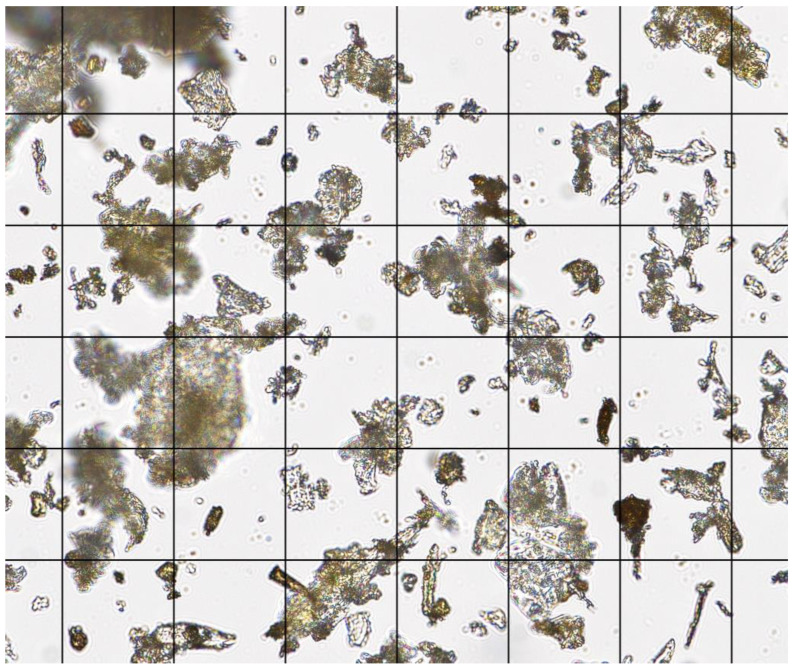
Image showing red oak dust particle shape and size on a 50-micron grid.

**Figure 10:**
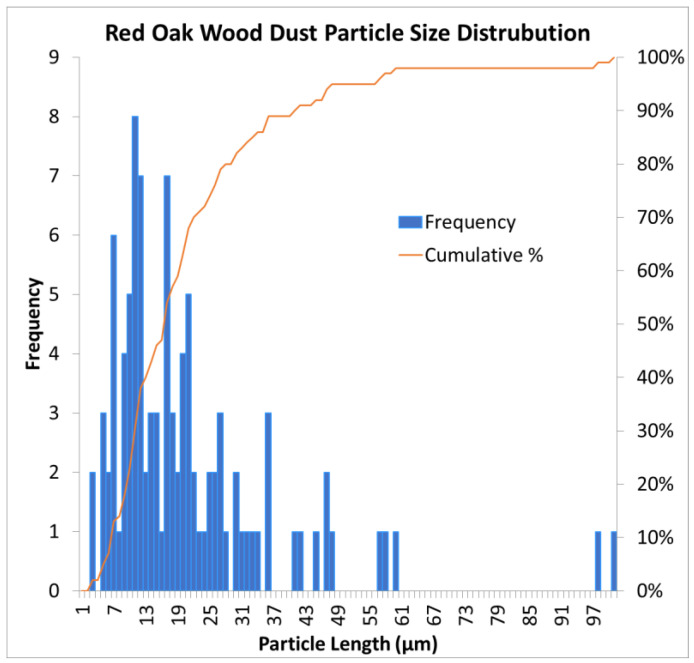
Red oak wood dust particle size distribution (*n* = 100 particles). Blue bars indicate frequency of each particle length, and the orange line indicates cumulative percentage.

Red oak wood was selected to study the effects of a single source of dust. Rough sawn red oak boards were purchased from a sawmill and reduced into approximately 12.7 mm (0.5 in.) wood chips using a chipper. The wood chips were bagged and transported to a controlled environment chamber at Penn State University where they were spread out on the floor and dried at 33 °C (92 °F) for 72 h. The dried wood chips were then transported to the Agricultural and Biological Engineering Building at Penn State University for further size reduction using a Munson SCC-10-MS rotary cutter (Munson Machinery Co., Utica, NY, USA) with a 1.6 mm (0.06 in.) diameter screen. The Munson grinder outlet had a chip collection basket at the bottom, and the top had a high-volume centrifugal dust collection system fitted with a fine mesh filter bag secured to a plastic 208-L (55 gallon) drum. Typical operation of the grinder involved collecting the coarse ground material and discarding the fine dust. However, the fine dust was needed, thus we collected fine dust from the 208 L (55 gallon) drum, then reprocessed the coarse particles through the grinder multiple times for further size reduction. The fine dust was then run through a mechanical sieve fitted with an 80-mesh filter cloth, and the fines were collected and bagged for use. The fine dust was placed on a metal pan and dried in an oven at 55 °C (131 °F) for 72 h to a moisture content of 4.8% (d.b.) before being used in the dust generator. Figure 9 shows a microscope image of the sieved red oak dust on a 50-micron grid. Figure 10 shows a graph of the sieved red oak dust particle size distribution. The mean particle length was 21 microns (*n* = 100 particles, min 2.1, max 160.6, SD 20.3).

During the six-week study, fine dust was brushed from all surfaces inside the controlled environment chamber and collected in a storage bin. The collected dust was then passed through a coarse sieve with 3.2 mm (0.13 in.) openings and 42% open area to capture feathers and very coarse material generated by the birds, followed by the 80-mesh filter cloth to separate coarse from fine dust. The fines were then oven dried for 72 h before being reused. A small volume of coarse dust particles was mixed with the fine dust to improve flowability of the powder and prevent packing. The coarse dust particles did not remain airborne but settled on the floor directly under the outlet within 2 m (6.4 ft) of the dust generator outlet.

### 2.9. Controlled Environment Chamber

**Figure 11:**
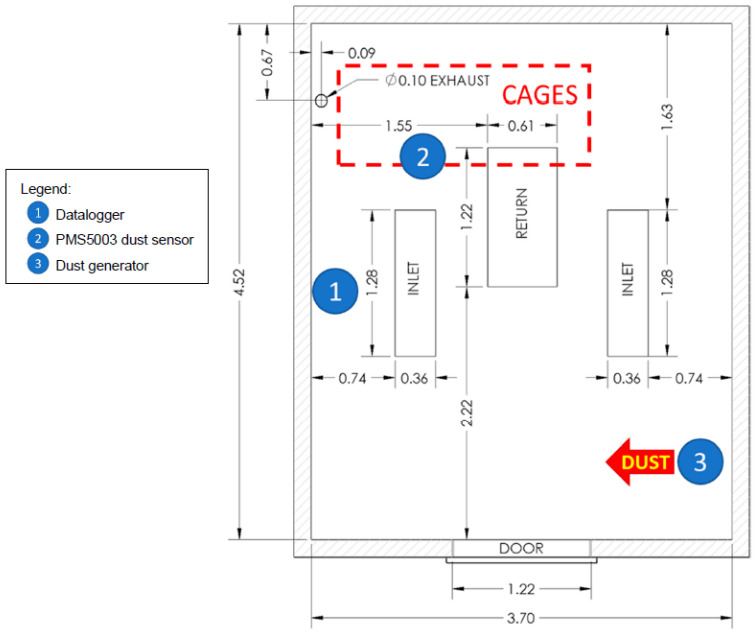
Plan view of controlled environment TEST chamber (all dimensions shown in meters). Location 1 = datalogger (0.3 m above floor), 2 = PMS5003 dust sensor (2 m above floor, on top of cages), 3 = dust generator (1 m above floor). Exhaust, inlet, and return openings were in the ceiling.

Experiments were performed in a controlled environment chamber (TEST chamber) at the Poultry Education and Research Center (PERC) at the Pennsylvania State University. The chamber had dimensions of 3.7 m × 4.3 m × 2.4 m (12 ft × 14 ft × 8 ft) and the walls, floor, and ceiling were stainless steel (Figure 11). Exhausted air was replaced by fresh outdoor air drawn into the room inlets by a continuously operating 0.06 m^3^/s (130 cfm) exhaust fan located in the ceiling in the back-left corner of the chamber. The air in the chamber was well mixed as a result of a 1.5 m^3^/s (3000 cfm) air handling unit that recirculated air in the room to maintain temperature and humidity settings. Temperature could be controlled between 4 °C and 40 °C (±0.5 °C), and humidity (at upper and lower temperature limits) from 60–90% at 4 °C, and from 25–80% at 40 °C. Humidity was added to the chamber by a Nortec EL-50 steam humidifier (Nortec Humidity Ltd., Ottawa, ON, Canada). The chamber dehumidifier was not operable during the experimental timeframe. Daily chamber maintenance was performed between 8:00 and 10:00 a.m. including brushing all equipment to remove excess dust, and cleaning chamber ventilation system air filters and ducting to remove accumulated dust.

**Figure 12:**
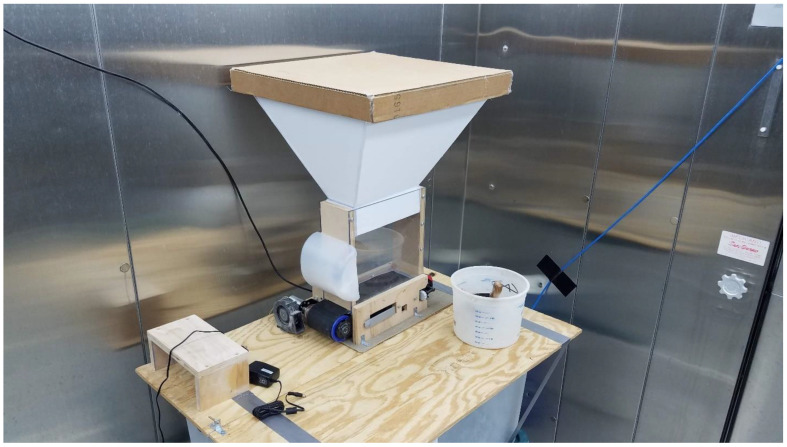
Dust generator placed in controlled environment chamber.

**Figure 13:**
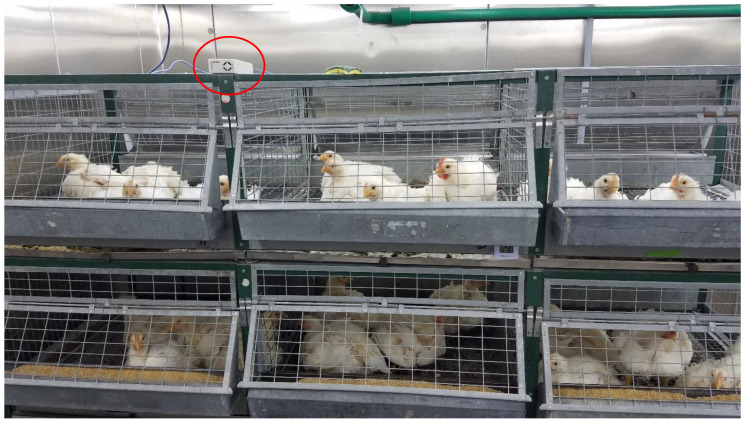
PMS5003 dust sensor enclosure (circled in red) placed on top of laying hen cages.

The dust generator was mounted to a plywood tabletop located in the controlled environment chamber in the front right corner opposite from the location of the ceiling exhaust opening (Figure 12). This location was used to give the dust more time to remain airborne before being removed by the ceiling exhaust. A four-level cage assembly was placed in the rear of the chamber, close to the room exhaust, to place birds between the dust generator and the exhaust opening with the goal of exposing the birds to airborne dust before it was removed from the air by the exhaust fan. The PMS5003 dust sensor enclosure was placed on top of the cages with the inlet and exhaust openings facing the door of the chamber (Figure 13).

Four birds were housed in each cage of the top two levels (total 24 birds). A second controlled environment chamber (CONTROL chamber) was set up in the same manner, but without dust generation so background levels of airborne dust in the chamber could be measured for comparison.

### 2.10. Dust Generator Usage and Evaluation

A six-week experiment was conducted using the dust generator to maintain a PM10 concentration of 50 µg/m^3^ in the TEST chamber for a poultry health study. The dust generator performance was evaluated during the last week of the experiment.

## 3. Results

The dust generator was operated continuously for six weeks to maintain a PM10 concentration of 50 µg/m^3^ in the TEST chamber for a poultry health study. The temperature inside the chamber started at 33.3 °C (92 °F) and was gradually reduced to 20.0 °C (68 °F) over the six-week period. The relative humidity level inside the chamber varied between 11 and 37% due to outdoor conditions (mean RH = 21.1 ± 6.5%). During the six-week experiment, the dust generator maintained an average PM10 concentration of 54.92 ± 6.42 µg/m^3^ (mean ± standard deviation, *N* = 517,122 measurements).

**Figure 14:**
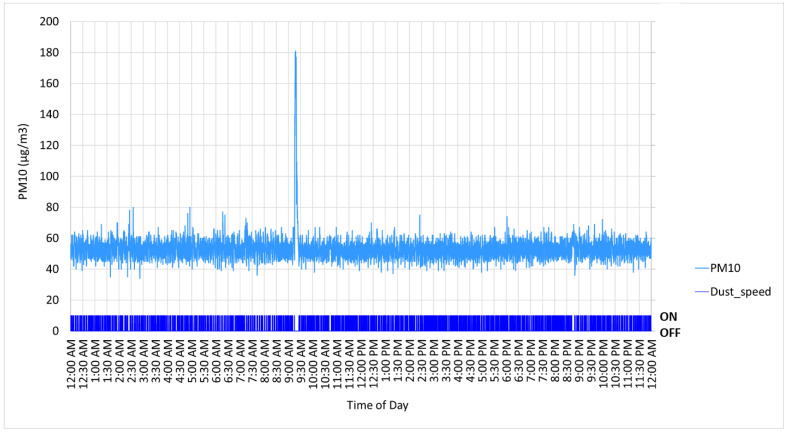
Measured PM10 concentration over 24-h period when dust generator was operating to maintain 50 µg/m^3^ in the chamber.

Figure 14 shows a graph of the measured airborne dust concentration inside the controlled environment chamber over a representative 24-h period during the last week of the experiment when the dust generator was operating to maintain an indicated PM10 concentration of 50 µg/m^3^. The spike in dust concentration at 9:15 am was due to daily chamber maintenance activities (cleaning the chamber ceiling air filters), so was considered an outlier and not included in statistical analysis. The dust generator maintained a PM10 concentration of 52.72 ± 4.68 µg/m^3^ (mean ± standard deviation, *N* = 14,835 measurements) over this 24-h period. For comparison, the measured dust concentration in the control chamber (only birds with no dust generated) was 5.67 ± 1.86 µg/m^3^ during the same 24-h period (*N* = 14,276). The total volume of red oak wood dust dispersed during that period was 8.7 L (529.2 in^3^). Particle count statistics for the test and control chambers are listed in Table 1. The control system sent the ON command to operate the dust generator 3772 times during the 24-h period (1 s on, 4 s off). This means the total daily run time for the dust generator was 1.05 h.

## 4. Discussion

### 4.1. Dust Generator Conveyor Motor Operation

During initial testing of the dust generator, simple ON-OFF operation of the dust generator was attempted. However, because each Arduino loop iteration took around 5 s, the dust generator would operate for 5 s each time the code sent a run command. This resulted in a large overshoot of the target level followed by a long period of off time. The Arduino code was changed to alter the duty cycle of the dust generator conveyor drive motor so it operated for only 1 s during each loop iteration. The reduced duty cycle produced acceptable results.

### 4.2. Advantages of Automatic Control

An advantage of the automatic control of the dust generator based on measured airborne particle concentration is the conveyor motor automatically operates for shorter or longer durations to maintain the setpoint dust concentration regardless of changes in: (1) material flow due to changes in humidity; (2) partial blockages in the outlet opening of the hopper resulting in a narrower trail of dust on top of the belt; (3) changes in dust particle size distribution of the material being used (i.e., if control is based on PM2.5 and more coarse than fine particles are in the material being discharged, the dust generator will operate longer to meet the fine particle concentration than if it were based on PM10). This gives the user much more flexibility in operation.

### 4.3. Control of the Dust Generator Output

PM10 was chosen as the control variable for dust generator output because it was not greatly affected by fine mist from the humidifiers used in the chambers under normal operating conditions, such as when the humidifiers run only enough to maintain a set humidity level. During testing the PM2.5, PM1.0, 0.3 µm, 0.5 µm, 1.0 µm, and 2.5 µm measurements from the PMS5003 laser particle counter were affected by water vapor. But the PM10, 5.0 µm, and 10.0 µm measurements were unchanged when the humidifier was operating.

### 4.4. Generated Dust Levels in Test Chamber Compared with Control

**Figure 15:**
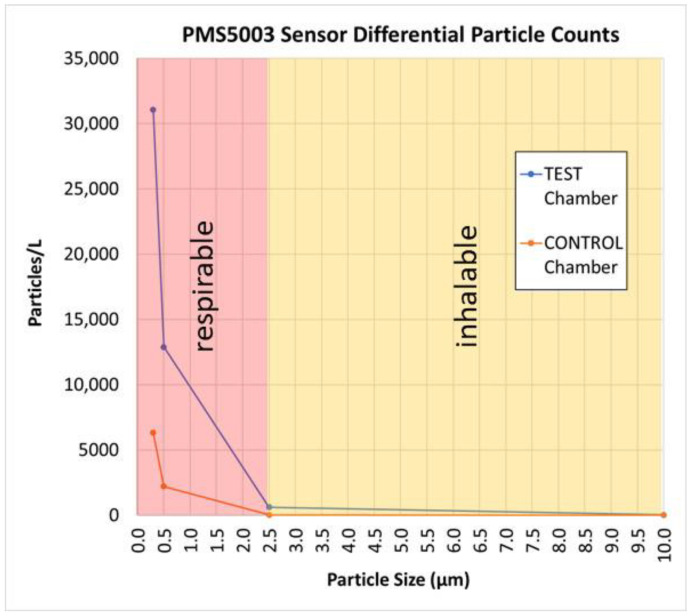
Graph showing measured 0.3, 0.5, 2.5, and 10 µm dust particle counts in the test and control chambers.

The control chamber dust particle counts listed in Table 1 were the background dust levels present in the chamber from bird feathers and dander, manure, feed, and incoming ventilation air. Figure 15 illustrates the test chamber had similar levels of inhalable dust (greater than 2.5 µm in diameter) compared to the control chamber, but the automatically controlled dust generator dispersed five to six times the number of respirable dust particles inside the test chamber when maintaining the setpoint concentration. This was more likely due to careful dust sample preparation (grinding, sieving, and drying) combined with settling characteristics of the red oak wood dust than the type of dust generator used.

### 4.5. Challenges

One of the biggest challenges faced when operating the dust generator was dust trapped by filters in the recirculation air handling system of the controlled environment chamber. A significant amount of dust was quickly trapped by the filters. In addition, some of the finest dust flowed past the filters and settled inside the ventilation ducting and accumulated on the air conditioning condenser. Daily cleaning of the chamber air filters was performed to remove trapped dust from the filters and to clean settled dust inside the ducting.

Another challenge is the laser particle counter used to automatically control the dust generator interprets smoke, steam, or fine mist as particulate matter. There was one event during early testing when maintenance was being performed on the chamber humidifiers, and the humidifiers were turned on to rapidly increase the indoor humidity level from 20 to 30%. During the rapid increase in relative humidity, which was achieved by injecting a high concentration of steam into the chamber air, the dust sensor readings increased to very high levels and the dust generator output was stopped because the dust sensor measured the injected steam as dust. However, after the humidity setpoint of 30% was reached, the steam output decreased greatly because the humidifier returned to normal operation, occasionally injecting low levels of steam to maintain a setpoint of 30%. The low concentration of steam did not interfere with the dust sensor PM10 measurements, and the dust sensor levels dropped back to normal levels and the dust generator continued to maintain the PM10 setpoint concentration of 50 µg/m^3^. If steady elevated levels of smoke, steam, or mist are expected, it may be necessary to recalibrate the laser particle counter under those conditions. It may also be possible to locate the laser particle counter farther from the source of mist or closer to the dust generator outlet to reduce interference from high background levels of mist.

### 4.6. Operation in High Humidity Environments

The automatically controlled dust generator was operated using sieved and dried red oak wood dust samples under conditions with low relative humidity to avoid problems arising from sawdust with a high moisture content. It is well known that dry wood is hygroscopic and will take on moisture in very humid environments. This is expected to cause problems for the dust generator if operated in high humidity environments, such as clumping, bridging, or reduced flowability of dust from the hopper. Extra care may be required to use dust generation equipment in the presence of high humidity, such as storing dust samples in a dry location in tightly sealed containers until use, and sealing the dust hopper lid to prevent moisture intrusion.

### 4.7. Dust Feed from Hopper

Initially, the vibratory shaker motor was run continuously while the conveyor belt gearmotor was operated intermittently. However, this caused two problems: (1) continuous operation resulted in rapid wear of the bushings in the vibrator motors, requiring them to be replaced after only three days of use; and (2) continuous operation of the vibratory motor when the conveyor belt was stationary resulted in consolidation and very tight packing of dust in the hopper, which stopped the flow of dust out of the hopper almost completely due to bridging. Intermittent operation of the vibratory motor only when the conveyor gearmotor was operating greatly reduced bushing wear and enabled dust to flow out of the hopper without causing bridging problems. A small volume of coarse dust particles was mixed with the fine dust to further improve flowability of the powder and prevent packing. The coarse dust particles did not remain airborne but settled on the floor directly under the outlet within 2 m (6.4 ft) of the dust generator outlet.

### 4.8. Conveyor Quality

We used the Ryobi belt sander frame and pulleys because it was inexpensive compared to a conveyor body fabricated from 80/20 aluminum channels and cast steel cogged pulleys. However, construction using these more expensive components would likely result in a more robust, longer lasting, and better tracking conveyor.

### 4.9. Dust Sensing Technology

Dust in animal housing is seldom uniform in shape or composition. Since optical particle counters cannot distinguish the actual composition of environmental dust being measured, it is important to use other sampling techniques such as gravimetric sampling [35] to double check measured dust concentrations. Sample analysis should be performed to characterize materials in the dust, shape and size of particles, and sample density so that concentration using optical particle counters can be accurately estimated. Handheld optical particle counters may be a good option for occasional spot checks of dust concentration, however, our experience has shown the sampling pump and dust sensor can be damaged when used in the presence of high levels of dust, so they may not be appropriate for long term measurements.

## 5. Conclusions

An inexpensive automatically controlled dust generator was developed and continuously tested for six weeks. The dust generator could generate and maintain a set airborne concentration of particulate matter based on measured real-time dust concentration.

## Figures and Tables

**Table 1:** Mean measured PM10 concentration and particle counts over 24-h period for test and control chambers.

Location		PM10	Average Particle Count, Particles/L					
		µg/m^3^	0.3 µm	0.5 µm	1.0 µm	2.5 µm	5.0 µm	10.0 µm
Test Chamber	Mean	52.72	44,617	13,537	3168	653	128	38
	SD ^1^	4.68	5736	1720	430	151	54	25
Control Chamber	Mean	5.67	8534	2227	315	27	9	4
	SD ^1^	1.86	1711	455	102	25	12	7

^1^ SD, standard deviation.

## Data Availability

Not applicable.

## Appendix A

Table A1 is a list of parts and electronic components used to build and control the dust generator.

**Table A1:** Parts list for automatically controlled dust generator and controller.

Quantity	Component
1	Ryobi BD4601G belt sander
1	375H400 timing belt
2	18-tooth 3D printed drive pulley half
2	18-tooth 3D printed idler pulley half
2	RJ45 jack (1 for generator, 1 for dust sensor)
2	RJ45 breakout board
1	12 VDC 1800 rpm vibratory shaker motor
1	5 VDC relay
1	Delta model BFB1012VH-7P03 centrifugal fan
1	9 VDC 1.5 A power adapter
1	3.5 RPM 12 VDC gearmotor
1	37 mm L-shaped motor mount for torque arm
6	18–8 stainless steel flanged button head screw, M3 × 0.5 mm thread size, 6 mm long
2	Flanged Philips head screws for torque arm stops
2	¼ in.–20 square nuts for torque arm stops
1	12 mm to 6 mm stainless steel set screw shaft coupler
1	L293N motor controller
1	3D printed motor controller housing
4	M3.5 × 6 mm thread forming screws
2	Female blade terminal plugs
1	2-pin JST-XH male plug
2	JST-XH female crimp pins
1	6-pin 2.54 mm pitch female connector housing
1	1-pin 2.5 mm pitch female connector housing
7	Female crimp pins
2	Plywood sides for conveyor hopper
2	Polycarbonate sides for hopper
2	UHMW polyethylene film adhesive-backed, 1 in. wide strip, 0.005 in. thick, 12 in. length for side skirting
1	UHMW polyethylene film adhesive-backed, 1 in. wide strip, 0.005 in. thick, 4 in. length for side skirting
1 box	Flanged Philips head screws
1 tube	E6000 adhesive
1	Slippery white UHMW polyethylene sheet, 4 in. × 12 in. × 1/16 in. for slider bed liner
4	1/8 in. diameter × 1/4 in. grip range medium steel pop rivets to attach liner to slider bed
1	Arduino MEGA 2560 R3 microcontroller
1	DS3231 real-time clock module
1	MicroSD card module
1	Plantower PMS5003 laser particle counter
1	3D printed dust sensor housing and lid
1	Polonium-210 disc source
1	LM2596 DC-DC voltage converter module
multiple	22-gauge silicone stranded wires
1 box	M2 × 4 mm thread forming screws
1 box	M3.5 × 6 mm thread forming screws
